# 1-DNJ Alleviates Obesity-Induced Testicular Inflammation in Mice Model by Inhibiting IKKβ/ NF-kB Pathway

**DOI:** 10.1007/s43032-024-01502-1

**Published:** 2024-03-07

**Authors:** Wenli Mai, Yi Shang, Yibin Wang, Ying Chen, Bo Mu, Qian Zheng, Hua Liu

**Affiliations:** 1 Institute of Basic Medicine and Forensic Medicine, North Sichuan Medical College, Sichuan, 637000 China; 2 The Second Affiliated Hospital of North Sichuan Medical College, Sichuan, 637000 China; 3 Department of Imaging Medicine, North Sichuan Medical College, Sichuan, 637000 China; 4 Department of Clinical Medicine, North Sichuan Medical College, Sichuan, 637000 China

**Keywords:** 1-DNJ, Obesity, Reproductive ability, Inflammatory factors, NF-kB

## Abstract

Obesity is associated with chronic inflammation that affects various organs in the body, including the reproductive system, which is a key factor in male infertility. 1-Deoxynojirimycin (1-DNJ) is a natural alkaloid in mulberry leaves, which has anti-inflammatory capabilities, yet, it’s effects on obesity-induced inflammation-related male infertility remain unclear. Therefore, this research investigates the underlying mechanism by which 1-DNJ may mitigate fertility impairment in male mice caused by obesity-related inflammation. Male mice with high-fat diet (HFD)-induced obesity were treated with 1-DNJ or metformin for 8 weeks. Metabolic profiles were evaluated by enzyme method. Reproductive capacity was assessed by sperm viability, motility and counts, immunohistochemistry was performed to evaluate the testicular damage caused by obesity and inflammation. The inflammation was assessed by measuring the levels of tumor necrosis factor α (TNFα), interleukin 1β (IL-1β), and interleukin 6 (IL-6). The activation of IκB kinase β (IKKβ) and nuclear factor κB (NF-κB) was examined using western blot and immunohistochemistry. HFD induced obesity in mice with obvious lipid metabolism disorder. The obese male mice had a decreased testosterone level, impaired sperm motility, and increased inflammatory factors. 1-DNJ treatment improved the testosterone level in the obese mice, ameliorated the testicular structure damage and improve sperm viability. In addition, 1-DNJ treatment inhibited IKKβ/NF-kB signaling pathway and reduced inflammation in obese mice. 1-DNJ can improve the fertility of obese men by reducing obesity as well as obesity-induced inflammation. These findings provide new insights for 1-DNJ to alleviate inflammation caused by obesity and provide future possibilities for treating male infertility.

## Introduction

As per the World Health Organization’s records, in 2016, over 1.9 billion adults worldwide were reported to be overweight, with more than 650 million categorized as obese [1]. Research data have shown that obese men have an increased risk of azoospermia or oligospermia, and body mass index (BMI) is negatively correlated with the quality and quantity of sperm [2, 3]. Obesity can reduce male serum testosterone levels and sperm quality [4, 5]. Furthermore, obesity is thought to be a systemic chronic inflammatory response resulted from adipocyte dysfunction [6]. Chronic inflammation caused by obesity can lead to decreased sperm motility, morphological defects, and DNA damage when inflammatory factors in semen increase [7, 8].

In obesity, chronic systemic low-grade inflammation is triggered by the release of numerous inflammatory factors [9], including TNF-α and IL-6, which can activate NF-κB pathway. The activation of NF-κB subsequently leads to the production of additional inflammatory mediators, perpetuating inflammation in a vicious cycle [10]. NF-κB is present in nearly all types of animal cells and serves as a crucial nuclear transcription factor to regulate the transcription of various genes [11], and is a key component of the classical inflammatory signaling pathway [12]. The activity of NF-κB is regulated by family of proteins known as IκBs (inhibitors of NF-κB). When in their inactivated state, NF-κB proteins are bound and inhibited by IκB proteins in the cytoplasm. Proinflammatory cytokines such as IL-1β and TNF-α [13, 14] can activate IKK complex (IKKβ, IKKα, and NEMO), which phosphorylates IκB proteins, leading to their degradation and freeing NF-κB. The IKK-β is the primary catalytic subunit of the IKK complex, and essential for activating NF-κB through typical or classical activation pathways [15–17]. The activated IKKβ exhibits Ser/Thr kinase activity directly phosphorylating IκBα at Ser32/36. This process releases the NF-κB heterodimer, primarily consisting of RelA (p65) and NF-κB1 (p50) subunits, enabling their translocation into the nucleus. Once inside the nucleus, the NF-κB heterodimers induce the transcription of target genes, ultimately activating many genes associated with pro-inflammatory cytokines and promoting the production of inflammatory factors [18]. Suleiman’s study further showed that the loss of testicular weight and the decrease of the number of reproductive cells in obese male mice were related to the increase of mRNA of inflammatory factors NF-κB, TNF-α and IL-1β in testicular tissue [19]. Therefore, we hypothesized that chronic inflammation caused by obesity may affect testicular spermatogenesis through the classical inflammatory pathway IKKβ/NF-κB, thereby impairing the reproductive ability of male obese mice.

Mulberry leaves have been used for a long time to treat male and female sexual reproductive function in Chinese traditional medicine. At present, mulberry leaves and their extracts are widely used to improve the reproductive capacity of female animals [20, 21]. Mulberry leaf extracts have been shown to reduce the consumption of testosterone in diabetic male rats [22]. 1-DNJ, one of the active ingredients in mulberry leaf extracts, is a type of piperidine polyhydroxy alkaloid [23] that is abundant in mulberry plants [24]. This compound exhibits various biological activities including antihyperglycemic [25, 26], lipid-lowering, and antitumor properties [27]. Previous studies have demonstrated that 1-DNJ effectively mitigates neuroinflammation [28] and septic cardiomyopathy [29] in SAMP8 mice. However, the potential application of 1-DNJ in addressing reproductive system inflammation associated with obesity has not yet been explored. In this study, we studied the effects of 1-DNJ on sperm quality and reproductive function in HFD-induced obese mice, and investigated its ability to regulate testicular inflammation and the underlying mechanisms involved.

## Materials and methods

### Antibodies and reagents

1-DNJ (purity ≥ 98%) (Catalog No. CSN16455) was purchased from csnpharm Co, Ltd (Shanghai, China;). Metformin (Catalog No. A2209016) was purchased from Yiling pharmaceutical Co, Ltd, Shijiazhuang, China. The ELISA kits for IL-6 (Catalog No. E-MSEL-M0003), IL-1β (E-MSEL-M0001), TNF-α (E-MSEL-M0002), testosterone (Catalog No. E-EL-0155C) were purchased from Elabscience Biotechnology Co, Ltd, Wuhan, China. The kit for microcolorimetry analysis of low-density lipoprotein cholesterol (LDL, Catalog No. E-BC-K221-M), high-density lipoprotein cholesterol (HDL, Catalog No. E-BC-K221-M), triglycerides (TG, Catalog No.E-BC-K261-M) were purchased from Elabscience Biotechnology Co, Ltd, Wuhan, China. Anti-p-NFκB antibody (Catalog No. YP0191) and anti-p-Ikkβ polyclonal antibody (Catalog No. YP0637) were purchased from ImmunoWay Biotechnology Company, SuZhou, China. NF-kB P65 (Catalog No. R0815) and Ikkβ (Catalog No.R1706-13) were purchased from Hangzhou Huaan Biotechnology Co., Ltd, China.

### Animals and experimental design

A total of 50 male C57BL/6N mice (16–19 g, 4 weeks old) were acquired from Beijing Vital River Laboratory Animal Technology Co, Ltd. (Beijing, China) under the license number SCXK (Beijing) 2019–0008. They were housed under regulated conditions (temperature: 22 ± 5 °C, relative humidity: 50 ± 10%, and a 12-h light/dark cycle) with ad libitum access to water and food. All animal procedures performed in this study adhered to the National Institutes of Health Guide for the Care and Use of Laboratory Animals and received approval from the Animal Ethics Committee of North Sichuan Medical College. The mice were randomly assigned to either the normal group(N, *n* = 10) with normal diet or the experimental group with high-fat diet (HFD, *n* = 40). The N group received a standard diet (ND), whereas the HFD group was given a HFD (D12492). Both the ND and HFD were supplied by Research Diets, Inc. Following an 8-week feeding period, mice with a body weight surpassing 120% of the average weight of the N group were deemed to satisfy the requirements for an obesity animal model [8]. Mice that fulfilled these criteria were further categorized based on their body weight and randomly assigned to one of four groups: Obesity model group (M, *n* = 10); Metformin (Yiling pharmaceutical Co, Ltd, Shijiazhuang, China; Catalog No. A2209016) group (Met, *n* = 10); DNJ low-dose group (DL, *n* = 10); and DNJ high-dose group (DH, *n* = 10). No significant differences in body weight were detected among the four groups (*P* > 0.05). The Met group was given metformin 150 mg/kg/d by intragastric administration, and DL and DH groups were given 1-DNJ 40 mg/kg/d and 80 mg/kg/d [28, 30], respectively, by intragastric administration for 8 weeks.

### Assessment of sperm count and motility

An epididymis from each mouse was placed in physiological saline. The epididymis was incised at the junction between the epididymal body and tail and gently squeezed. One epididymis was then transferred to 35 °C physiological saline, divided into three sections, and carefully pressed to extract semen from the vas deferens, allowing it to mix with the saline. Sperm count per microliter (15 μL on each side) was ascertained using a hemocytometer. Sperm count and motility were evaluated per the WHO guidelines (fifth edition), with at least 200 sperms counted per sample. Sperm motility was graded as follows: grade 0—immotile; grade I—non-progressive or spinning in place; grade II—slow forward movement or forward rotation; grade III—rapid forward movement in an arc; and grade IV—rapid straight forward movement. Sperm motility (%) was calculated as (III + IV)/(0 + I + II + III + IV) × 100% [8].

### Micro colorimetry

LDL, HDL or TG in serum were determined via micro colorimetric assays according to the manufacturer’s instructions. In brief, 5ul of double distilled water, standard solution, or serum to be tested was added into the blank well, standard well, and sample well, respectively, incubated with 180ul reagent at 37℃ for 5 min. The optical density (OD value) for each well was determined with a micro-plate reader with a wavelength of 546 nm, and the OD value was recorded as A1. 60ul reagent 2 were added to the above and incubated at 37℃ for 5 min. OD values were determined with a micro-plate reader set to 546 nm, and were recorded as A2 [31]. Calculate the result according to the A1 and A2. Aspartate aminotransferase (AST), alanine aminotransferas (ALT), blood urea nitrogen (BUN), creatinine **(**CRE) were detected in the second affiliated hospital of North Sichuan Medical College.

### ELISA

Serum levels of IL-6, IL-1β, and TNF-α were assessed using ELISA kits, according to the manufacturer’s instructions. Samples or standards (100 µl) were added to each well of a microplate precoated with biotinylated antibody specific to IL-6, IL-1β,TNF‑α and incubated at 37℃ for 90 min, following by incubation with 100μL of biotinylated antibody working solution at 37℃ for 60 min. After washing, 100 μL of enzyme conjugate working solution was added to each well and incubate at 37℃ for 30 min. After washing, TMB was added to each well (90 µl/well) and incubated for 15 min at 37℃ in the dark following by addition of 50 μL of Stop Solution. Absorbance at 450 nm (OD value) was determined with a micro-plate reader. The values for serum samples were calculated based on the standard curve. Serum testosterone concentrations were measured similarly using an ELISA kit, following the manufacturer’s instructions [32].

### Hematoxylin–eosin staining

Tissue specimens were dehydrated through a series of graded ethanol, embedded in paraffin, and sliced into 5 μm thick sections using a microtome (Leica, USA). After deparaffinization, the sections were rehydrated with graded ethanol, stained with Hematoxylin for 1 min, differentiated with hydrochloric acid alcohol, rinsed with distilled water, soaked with eosinsolution for 20 s [33].

### Immunohistochemistry

4% paraformaldehyde-fixed testicular tissue underwent dehydration using a graded ethanol series, followed by clearance in xylene, embedding in paraffin, and sequential sectioning at a thickness of 5 μm. After deparaffinization, rehydration with graded ethanol, and endogenous peroxidase removal by incubation with hydrogen peroxide at 37 °C for 10 min, the sections were treated with citrate buffer at 100 °C for 15 min, and incubated with anti-p-NFκB antibody or anti-p-Ikkβ polyclonal antibody, were added, and sections overnight at 4 °C. After washing with PBS three times, the secondary antibody was added dropwise, and the immunohistochemical reaction was amplified using streptavidin–biotin complex (SABC). Chromogenic development was carried out using 3'-3-diaminobenzidine (DAB), while hematoxylin was employed for counterstaining. Image J software was utilized for analysis [34].

### Western blotting

Testicular tissue underwent homogenization in RIPA lysis buffer inclusive of phosphatase and protease inhibitors on ice. Total proteins were isolated using sodium dodecyl sulfate–polyacrylamide gel electrophoresis and transferred onto polyvinylidene fluoride membranes. After blocking with 5% nonfat milk, membranes were incubated with antibodies against p-NFκB, NF-kB P65, p-Ikkβ and Ikkβ at 4 °C overnight. Following washing with TBST, the membranes were incubated at room temperature for 1 h with secondary antibodies conjugated to horseradish peroxidase. An enhanced chemiluminescence reagent was added, and film exposure was performed in a darkroom [33].

### Statistical analysis

Variations between the acquired values (mean ± SD) were evaluated using one-way analysis of variance or independent sample t-tests, followed by post hoc least significant difference multiple comparison tests. A *p*-value < 0.05 was deemed to be statistically significant.

## Results

### 1-DNJ reduces the weight of obese mice

**Fig. 1:**
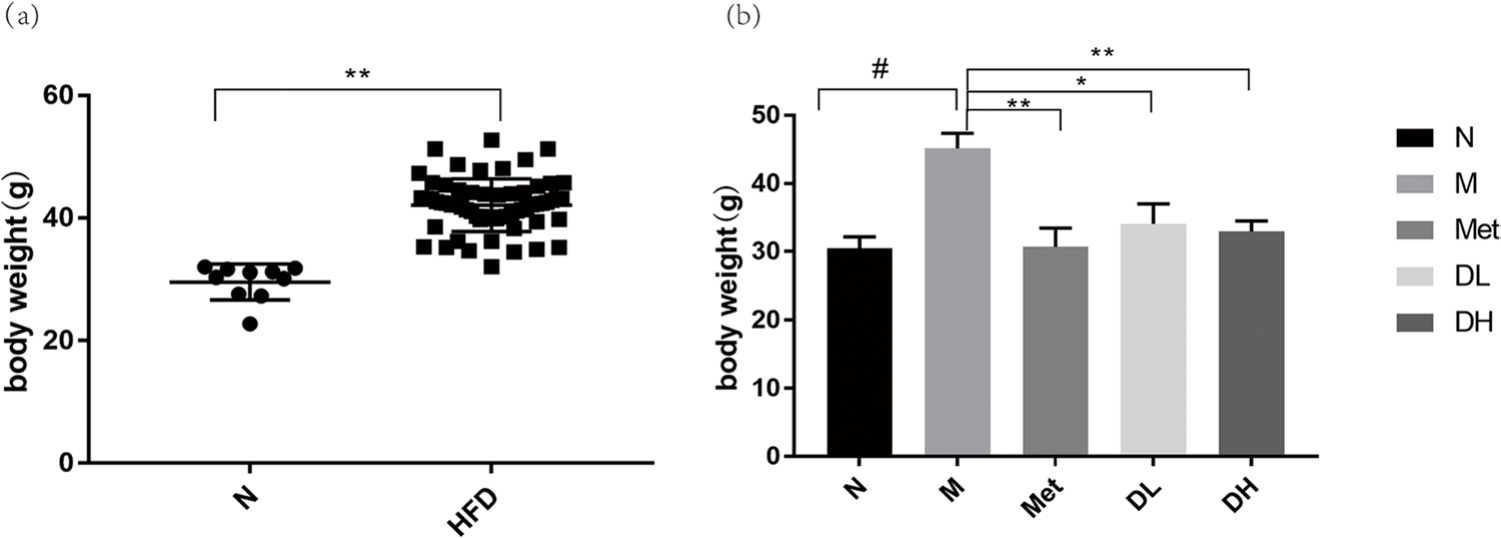
Impact of HFD and 1-DNJ on body weight. Weight of the mice after 8 weeks of feeding with normal diet (N, *n* = 10) or high-fat diet (HFD, *n* = 40) (**a**). Weight of control mice with normal diet (N, *n* = 10), or Obesity model group (M, *n* = 10) or HFD fed mice after 8 weeks of treatment with metformin (Met), low dose (DL) or high dose (DH) of 1-DNJ (**b**). Data are shown as mean ± SE. vs. N:^##^*P* < 0.01; vs. M:^*^ *P* < 0.05, ^* *^ P < 0.01

After consuming HFD for 8 weeks, the body weight (Fig. 1(a)) in HFD group exhibited a significant increase compared to the N group. The average body weight was 42.05 g and 29.58 g for HDF mice and normal control mice, respectively. The HFD group was 120% higher than that of N group, which confirmed the successful establishment of an obese mouse model induced by an HFD. Following another 8-week of DNJ treatment, both low (DL) and high dose (DH) of DNJ notably reduced the body weight (Fig. 1(b)) compared to Obesity model group (M). Metformin (Met) has been known to reduce weight [35–37] and used as a positive control. Surprisingly, both Met and DH groups showing a more substantial decrease than the DL group (Fig. 1(b)).

### 1-DNJ reduces glucose and lipid metabolism in obese mice

**Fig. 2:**
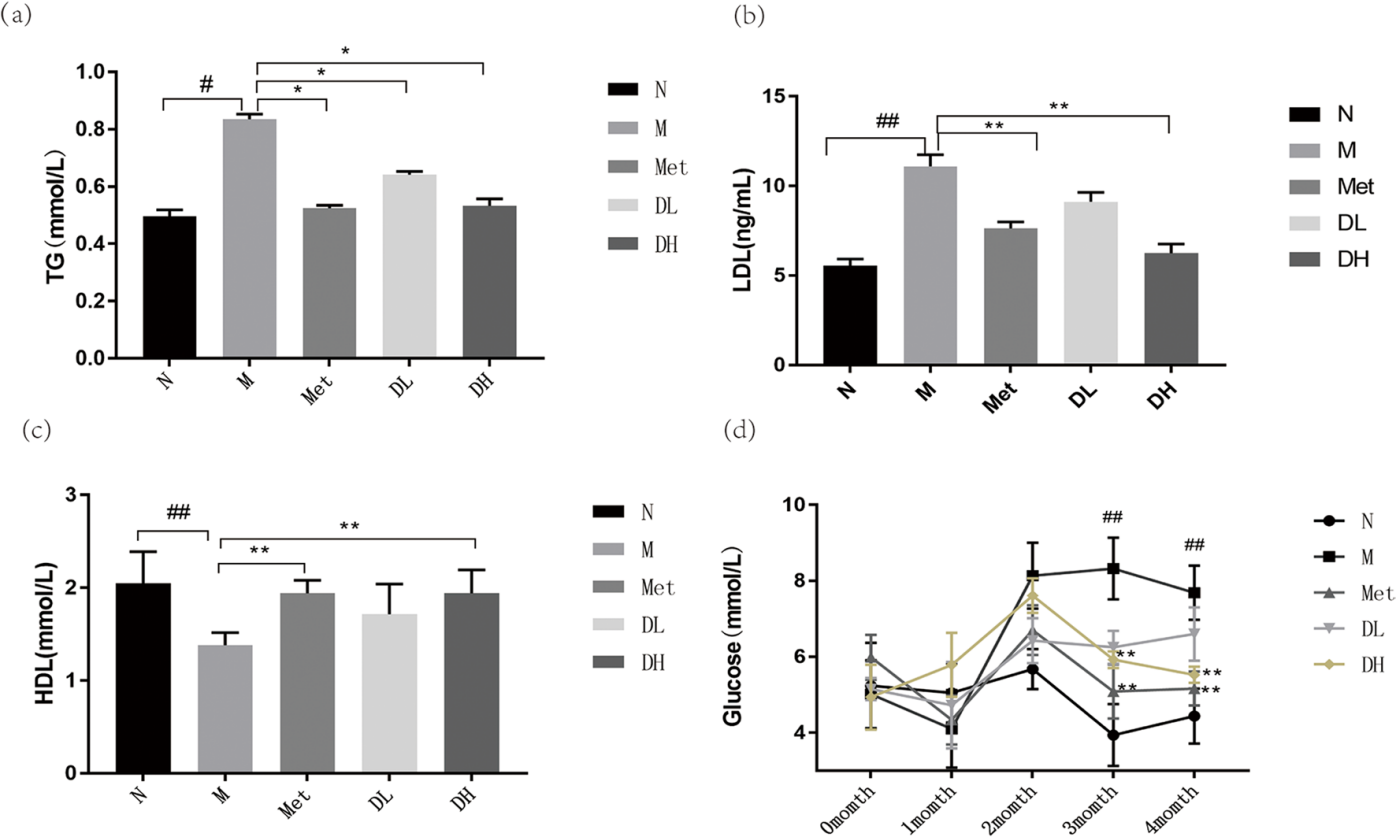
Impact of HFD and 1-DNJ on glucose and lipid Metabolism. After 8 weeks of treatment with metformin or 1-DNJ, the levels of TG (**a**), LDL (**b**), and HDL (**c**) in serum of mice were measured. Fasting blood glucose in mice fed with normal (N) or high-fat feeding (M). Eight week later, the HDF mice were treated with metformin (Met), low dose (DL) or high dose (DH) of 1-DNJ (**d**). Data are mean ± SE; N: normal control; M: Obesity model group; Met: Metformin group; DL: DNJ low dose group; DH: DNJ high dose group, vs. N:^##^*P* < 0.01; vs. M:^*^ *P* < 0.05, ^* *^ P < 0.01

Obesity is frequently associated with dyslipidemia [38], which is characterized by elevated plasma TG and LDL levels or reduced plasma HDL levels [38, 39], as well as insulin resistance or hyperglycemia [40]. Therefore, we measured the serum lipid profiles and glucose levels in the obese mouse model. We found that TG and LDL levels in the M group were significantly higher compared to those in the N group (Fig. 2(a) and (b)). More importantly, treatment of Met or DNJ significantly reduced TG and LDL levels, even with low dose of DNJ (DL group). By contrast, the HDL level in the M group was significantly lower in the N group (Fig. 2(c)), whereas was increased by metformin or DNJ treatment. As the feeding weeks increased, blood glucose levels in the M group rose (Fig. 2(d)), whereas those in the Met, DL, and DH groups declined after drug administration.

### 1-DNJ reduces plasma testosterone and inflammatory markers

**Fig. 3:**
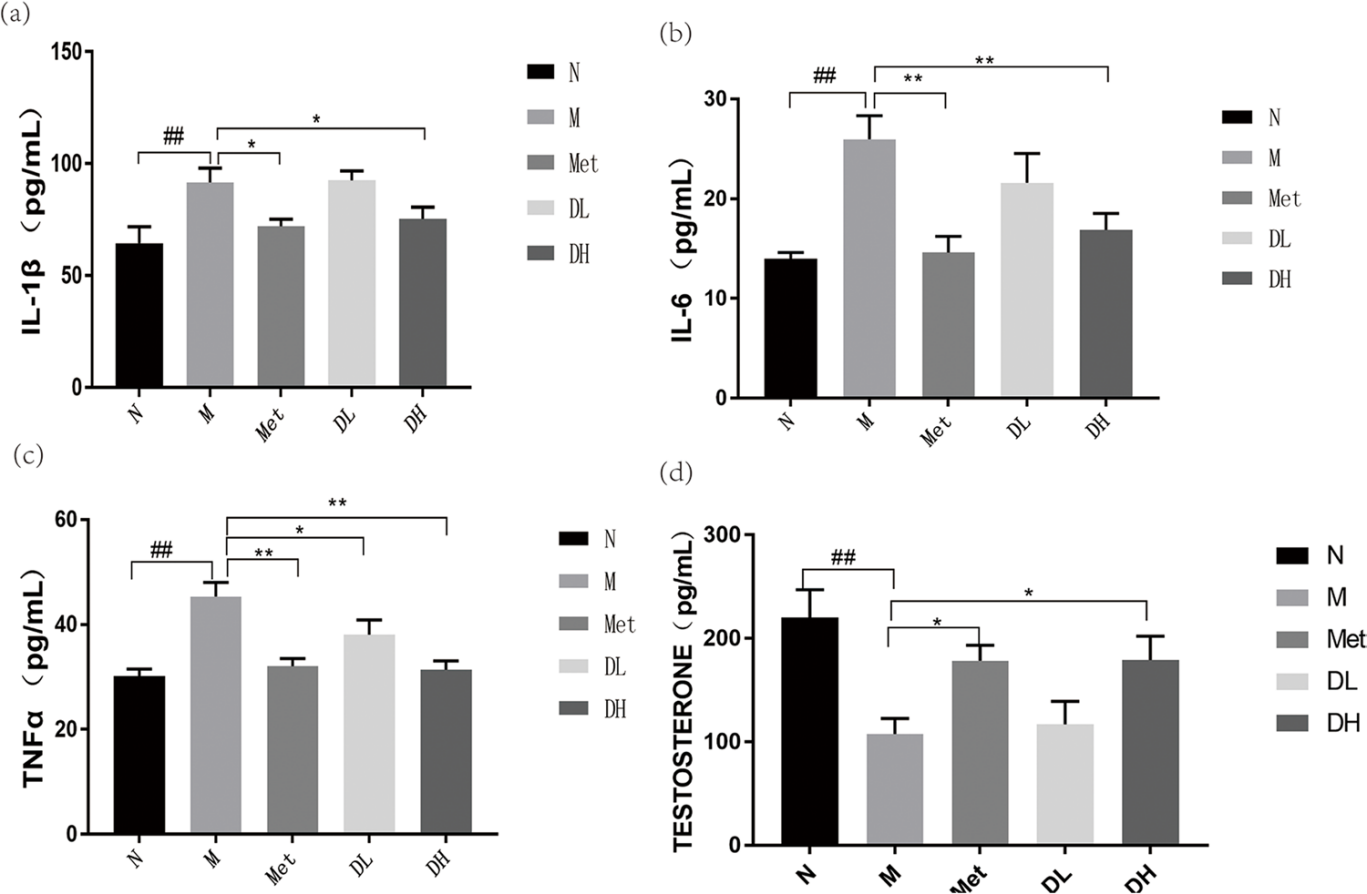
Impact of HFD and 1-DNJ on proinflammatory cytokines or testosterone in plasma. After 8 weeks of treatment with metformin or 1-DNJ, the levels of IL-1β (**a**), IL-6 (**b**), TNFɑ (**c**), testosterone (**d**) in serum were measured. Data shown are mean ± SE; N: normal control; M: Obesity model group; Met: Metformin group; DL: DNJ low dose group; DH: DNJ high dose group, vs. N:^##^*P* < 0.01; vs. M:^*^ *P* < 0.05, ^* *^ P < 0.01

Obesity-related chronic inflammation is characterized by the fact that hypertrophic adipocytes and secretion of inflammatory adipokines and cytokines [41, 42]. Therefore we investigated blood inflammatory cytokines in the mice. As expected, there were significant increases of IL-1β, IL-6, and TNF-α in plasma from mice in the M group compared to that from N group (Fig. 3(a), (b), and (c)). Notably, following an 8-week drug intervention, the IL-1β, IL-6, and TNF-α in plasma of the Met, DL, and DH groups significantly decreased. In contrast, testosterone levels were decreased in M group but rescued by metformin or DNJ treatment (Fig. 3(d)).

### 1-DNJ does not damage the metabolic function of liver and kidney in obese mice

**Fig. 4:**
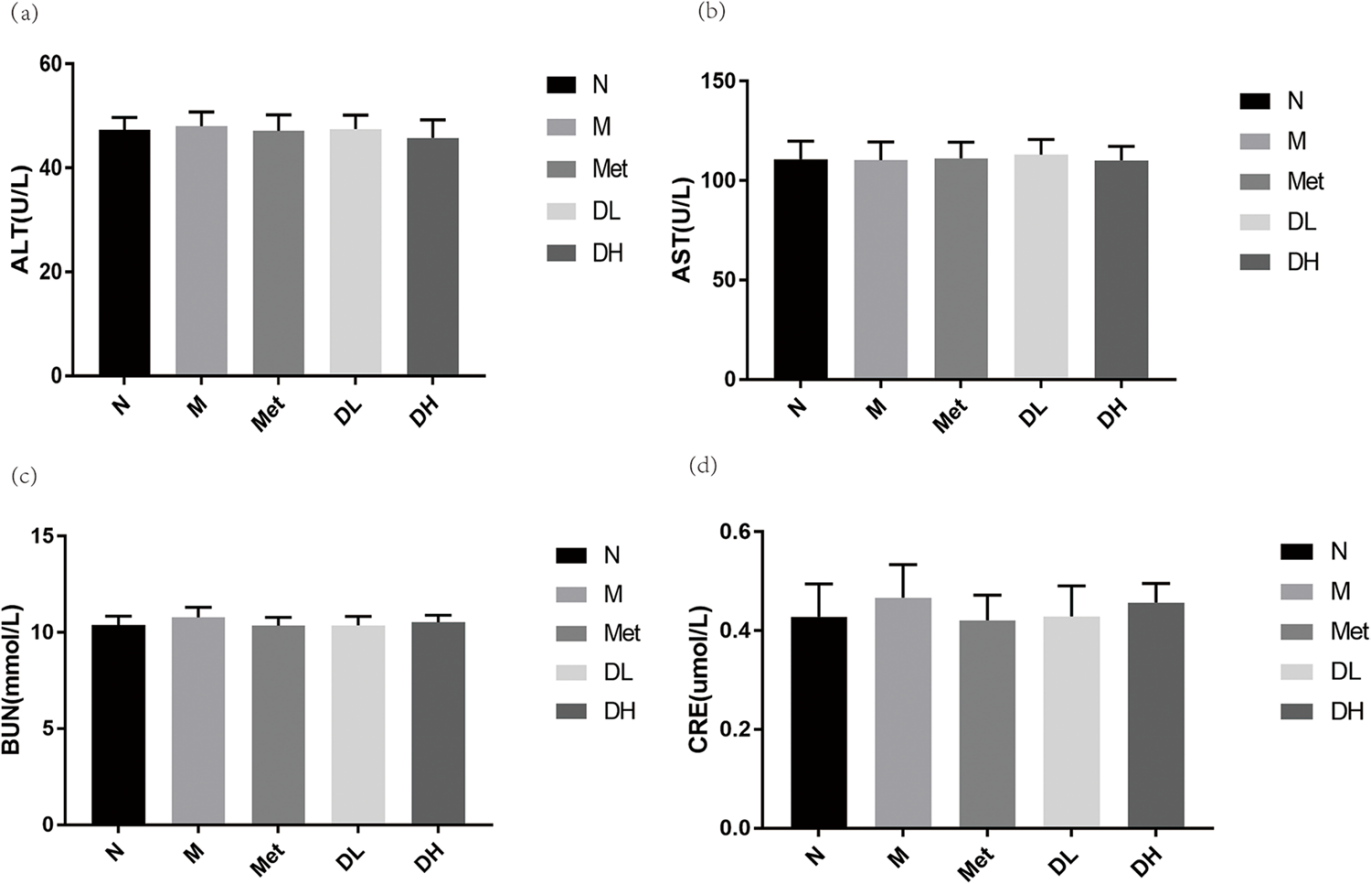
Impact of HFD and 1-DNJ on plasma AST, ALT, BUN, and CRE. After 8 weeks of treatment with metformin or 1-DNJ, the levels of ALT (**a**), AST (**b**), BUN (**c**), CRE (**d**) In serum were measured. Data shown are mean ± SE; N: normal control; M: Obesity model group; Met: Metformin group; DL: DNJ low dose group; DH: DNJ high dose group

After 8 weeks of administration, there were no differences in ALT and AST levels in the serum of mice indicating DNJ, even with high doses has no harmful effects on liver or kidney (Fig. 4(a) and (b)). Likewise, there were no differences in CRE and BUN levels in the serum of mice in each group (Fig. 4(c) and (d)).

### 1-DNJ can protect testicular structure and increase sperm motility in obese mice

**Fig. 5:**
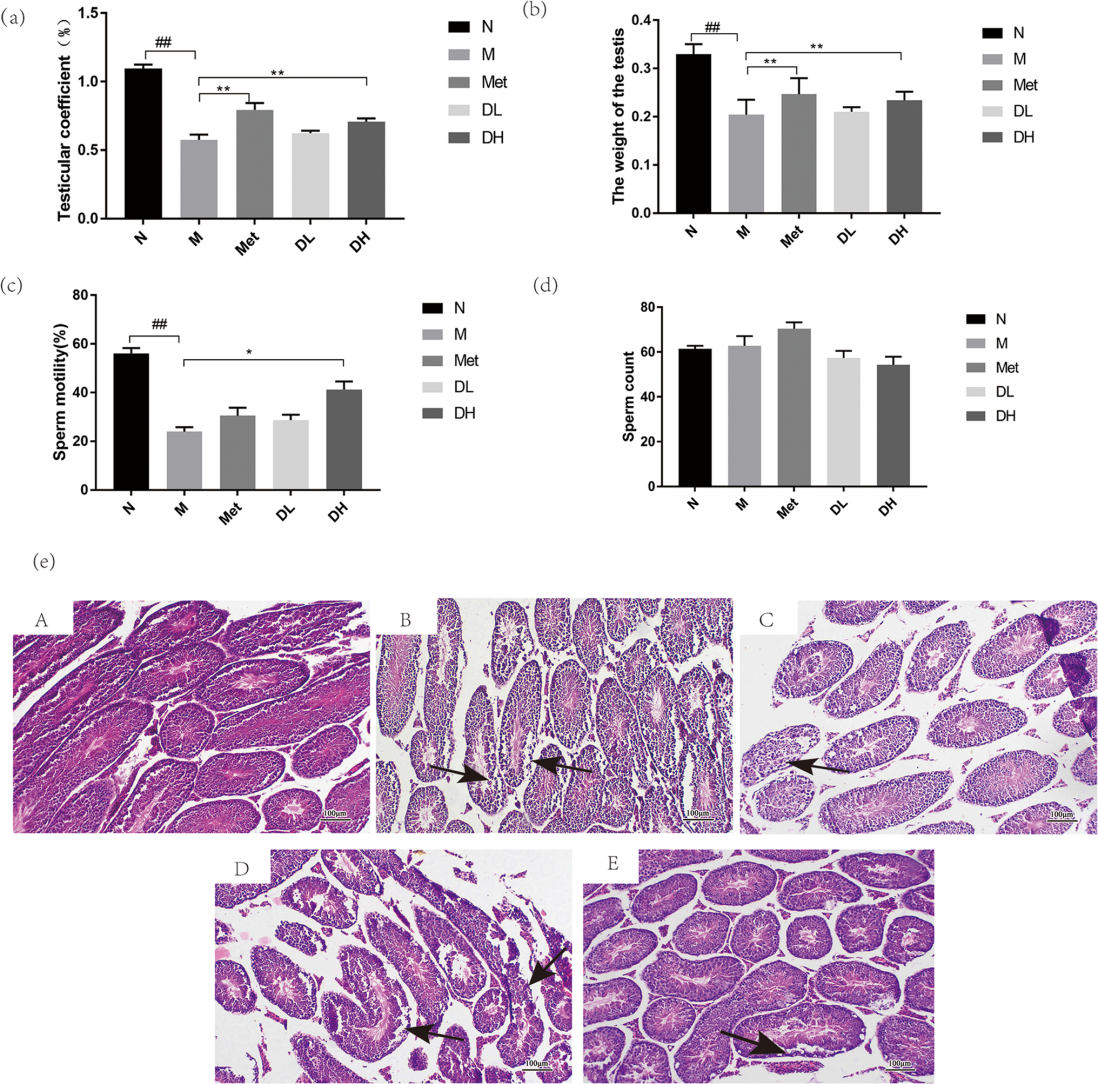
The protective effect of 1-DNJ on testis and sperm. After 8 weeks of treatment with metformin or 1-DNJ, the testicular coefficient (**a**), testicular weight (**b**) and,the sperm motility (**c**) and sperm counts(**d**) of mice in each group. After 8 weeks of treatment with metformin or 1-DNJ,HE staining of the testis (**e**). A: normal control; B: Obesity model group; C: Metformin group; D: DNJ low dose group; E: DNJ high dose group. Representative images at 100 × magnification; bar indicates 100um. *n* = 5. N: normal control; M: Obesity model group; Met: Metformin group; DL: DNJ low dose group; DH: DNJ high dose group.vs. N:^##^*P* < 0.01; vs. M:^*^ *P* < 0.05, ^* *^ P < 0.01

Testicular quality and testicular coefficient in male mice fed an HFD were notably lower compared to the N group. After drug administration, both testicular weight and testicular coefficient in the Met and DH groups substantially increased in comparison to the M group (Fig. 5(a) and (b)). There is no significant difference in in sperm count between N group and other group’s mice (Fig. 5(d)). Although the sperm count in M group and Met group is slightly higher than that in normal group, most were dead sperm (Fig. 5(d)). Compared with N group, the sperm motility of obese mice in M group decreased significantly. There was a slight increase in sperm motility in Met mice compared to the M group, but the difference was not significant (Fig. 5(c)). Interestingly, treatment with high-dose DNJ significantly increased the sperm motility (DH) as compared with group M (Fig. 5(c)). Furthermore, we evaluated the testicular histological sections in these mice Fig. 5(e). Tissue sections from the control group (N) showed a typical testicular architecture when stained with HE. However, there were some abnormalities in the mice from M group, including widened testicular interstitium, a relatively large lumen, reduced number and layers of spermatogenic cells, or irregular arrangement, or arrangement breaks. The histological structure of testes in obese mice treated with a high dose of 1-DNJ or metformin closely resembled that of the normal mice, while testes from mice treated with a low dose of 1-DNJ displayed partial breaks and irregular arrangement.

### 1-DNJ alleviates testicular inflammation by affecting IKKβ/NF-κB pathway

**Fig. 6:**
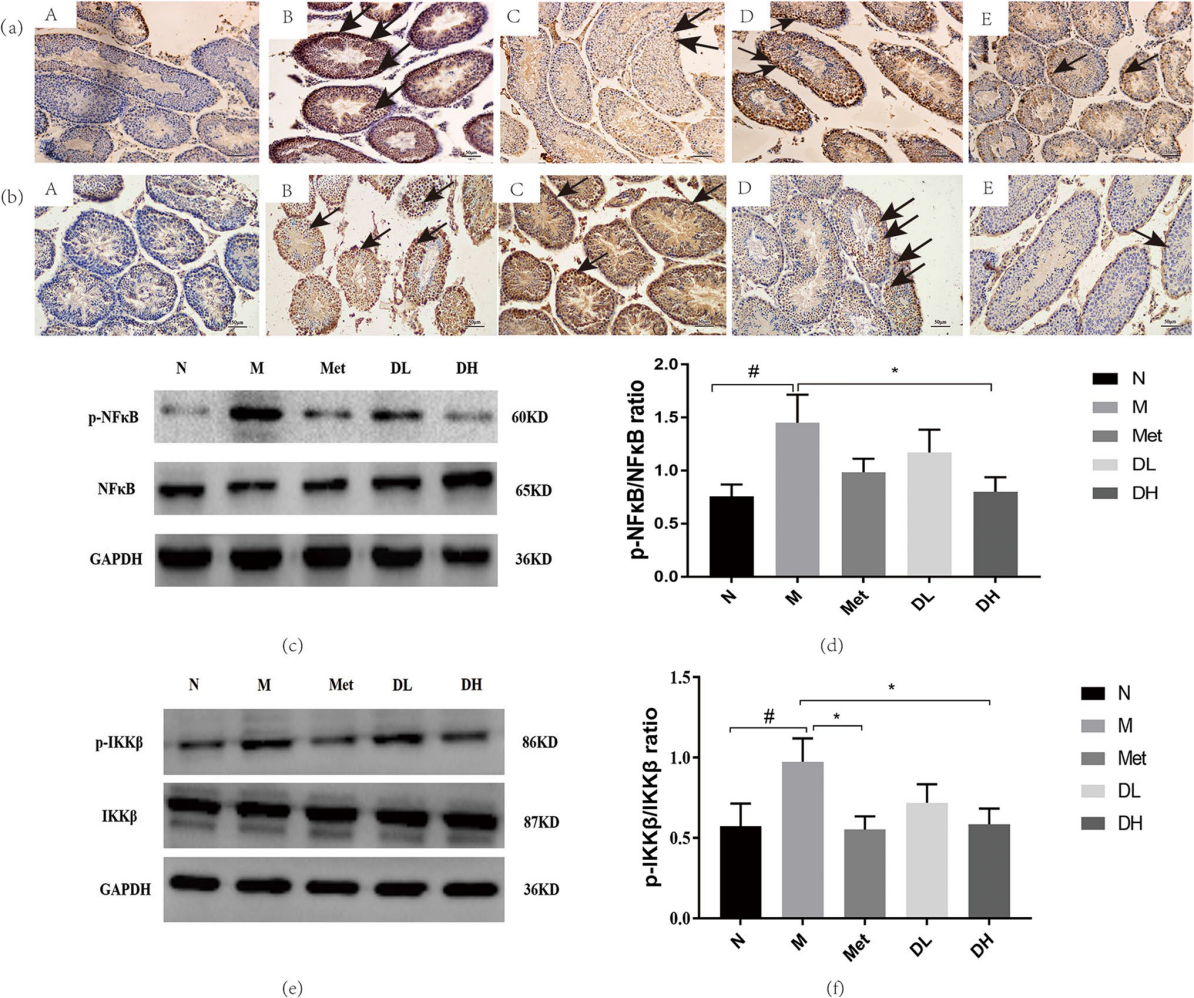
Effects of 1-DNJ on testis pIKKβ and pNF-κB. (**a**) Immunohistochemical staining of pIKKβ (**a**) or pNF-κB (**b**) in mouse. A: normal control; B: Obesity model group; C: Metformin group; D: DNJ low dose group; E: DNJ high dose group. Representative images at 200 × magnification; bar indicates 100um. (*n* = 5). (**c**-**e**), After 8 weeks of treatment with metformin and 1-DNJ, the expression of pIKKβ (**c**, **d**) and pNF-κB (**e**, **f**) in testis were detected by WB. **c** and **e** Data are mean ± SE (*n* = 5); N: normal control; M: Obesity model group; Met: Metformin group; DL: DNJ low dose group; DH: DNJ high dose group, vs. N:^##^*P* < 0.01; vs. M:^*^ *P* < 0.05

IKKβ is essential for canonical NF-κB activation in response to proinflammatory cytokines [43, 44]. Higher immunostaining intensity for pIKKβ and pNF-κB was observed in the testes from the mice in the M group compared to the N group. pIKKβ (Fig. 6(a)) and pNF-κB (Fig. 6(b)) proteins were distributed in round of sperm cells, spermatogonia, Leydig cells, and Sertoli cells in the testes of obese mice. There was a significant reduction in integrated optical density (IOD) of pIKKβ and pNF-κB in the testes of high-dose 1-DNJ or metformin-treated mice compared to untreated obese mice in the M group. Moreover, the positive expression of pIKKβ and pNF-κB in the DH group was significantly lower than in the DL group, indicating a dose effect. Similarly, western blot results showed increased phosphorylated IKK-β (Fig. 6(c,d)) and NF-κB (Fig. 6(e,f)) in the testes of mice in the M group as compared to that in mice from the normal group. More importantly, high-dose of 1-DNJ or metformin treatments effectively reduced phosphorylated IKK-β and NF-κB.

## Discussion

With the improvement in living conditions, individuals are consuming a higher amount of high-fat and high-calorie foods, leading to an increase in the obese population [45]. Male infertility associated with obesity has dramatically risen worldwide [46] and has also received much significant attention from the medical community [47, 48].The mechanisms through which obesity affects male reproductive function are complex. Research by Hotamisligil and colleagues suggests that obesity is a systemic state of chronic low-grade inflammation, with the resulting inflammatory response believed to be a key risk factor for obesity-related complications [9].This chronic low-grade inflammatory state associated with obesity is closely linked to the release and induction of numerous related inflammatory factors [4].TNF-α and IL-6 are inflammatory cytokines associated with obesity, particularly abdominal obesity [49]. For instance, elevated levels of IL-6 have been positively correlated with the expansion of adipose tissue [50].TNF-α, IL-6, and IL-1β can enhance the inflammatory cascade reaction and promote inflammatory responses [49], which are considered key triggering factors for obesity-related complications. Reports have indicated the detection of high levels of IL-6, TNF-α, and NF-κB in the reproductive tissue of SD obese rats and C57BL/6 obese mice fed a high-fat diet, along with reduced testosterone levels, thereby affecting spermatogenesis [31, 32].Therefore, we hypothesize that obesity-induced chronic inflammation is indeed related to damage in the male reproductive system. In this study, our research team successfully established an obese male mouse model by inducing a high-fat diet (HFD) in C57BL/6N mice. We analyzed the levels of IL-1β, IL-6, and TNF-α in mouse serum. The results showed a significant increase in the levels of these inflammatory factors in the serum of mice on a high-fat diet. Additionally, by HE staining, we observed that obese male mice exhibited widened testicular interstitium, relatively larger tubule lumen, and reduced numbers of spermatogenic cells. The spermatogenic cells diminished in cells counts and layers, exhibiting irregular or disrupted arrangements. The structural damage to the testes also led to a decrease in testicular weight and testicular coefficient. At the same time, testicular structural damage resulted in a significant decrease in testosterone levels and sperm vitality in obese mice. These findings are consistent with previous research [51, 52].Therefore, targeting the treatment of chronic inflammation in the reproductive system induced by obesity is an effective approach to improving the reproductive capacity of obese males.

In traditional Chinese medicine, mulberry leaves are commonly used to regulate reproductive system hormone levels and their anti-inflammatory properties [20, 21]. 1-DNJ (1-Deoxynojirimycin) is a unique natural active component found in mulberry sericulture products, primarily present in the branches, leaves, and roots of the mulberry tree. It functions as an α-glucosidase inhibitor and was previously used for its glucose-lowering effects in diabetes [53, 54].As research progressed, it was discovered that 1-DNJ could reduce liver inflammation and protect the liver in db/db mice [30]. It has also been found effective in alleviating neuroinflammation in SAMP8 mice [28] and septic cardiomyopathy [29]. However, there have been no reports of the use of 1-DNJ in the treatment of reproductive system inflammation. In this study, obese mice were orally administered 1-DNJ for 8 weeks, and the reproductive parameters of the mice were observed. The results showed that in the 1-DNJ treatment group, the testicular weight and sperm vitality of the mice increased, especially in the high-dose 1-DNJ treatment group, where serum levels of IL-1β, IL-6, and TNF-α significantly decreased. The testicular coefficient improved, the testicular interstitium resembled that of the normal group, and there was no significant enlargement of the tubule lumen. The number and layers of spermatogenic cells increased, indicating a normal arrangement. Sperm vitality and testosterone levels also significantly increased compared to the control group (M group). These results suggest that 1-DNJ effectively reduces inflammation caused by obesity, thereby protecting testicular function and improving sperm quality. To explore the mechanisms of 1-DNJ’s anti-inflammatory effects, our research team conducted a study on the inflammatory signaling pathways.

IKKβ/NF-κB is a classical pathway of inflammation. In the classical signal pathway involving NF-κB, IKKβ is phosphorylated by inflammatory factors, and IKKβ subsequently phosphorylates the inhibitor of NF-κB (IκB)α [55]. This process leads to the translocation of the NF-κB/Rel dimer into the cell nucleus, thereby regulating the expression of genes involved in inflammation [17]. In this experiment, we investigated whether the action of 1-DNJ in reducing serum inflammatory factors operates through the IKKβ/NF-kB pathway. We further studied IKKβ and NF-kB in the testes of mice. The results showed a significant increase in the levels of pIKKβ and pNF-κB in the testes of obese mice. After treatment with 1-DNJ, Western blot and immunohistochemistry results showed a decrease in the levels of pIKKβ and pNF-κB. Combining these changes with alterations in serum inflammatory factors, it is evident that 1-DNJ can inhibit the classical pathway of inflammation by reducing the phosphorylation of IKKβ and NF-κB, reducing the production of inflammatory factors in obese individuals, and mitigating the vicious cycle of inflammation [10]. In our research, we also observed a dose-dependent pharmacological effect of 1-DNJ. High-dose 1-DNJ significantly inhibited the phosphorylation of IKKβ and NF-κB, reducing the levels of inflammatory factors in the serum. In contrast, the DL group did not exhibit significant differences from the M group in the results mentioned. This also led to structural damage in the testicular tissue of the DL group mice and a decrease in sperm vitality, while the DH group mice showed improved testicular structure and function, along with higher sperm vitality. Therefore, it can be concluded that the pharmacological effects of high-dose 1-DNJ are superior to those of the low-dose.

Metformin is a classic antihyperglycemic agent [35–37, 56]. Recent research has shown that Metformin can also improve the fertility of obese males by reducing blood-testis barrier (BTB) damage caused by oxidative stress [46]. Additionally, during the inflammation process, Metformin can decrease the expression of pro-inflammatory factors such as TNF-α, IL-6, and IL-1β while increasing the expression of the anti-inflammatory factor IL-10. Metformin can also alleviate inflammation by inhibiting the activation of the IKKβ/NF-κB signaling pathway [57–59]. In our study, Metformin indeed reduced inflammatory factors in the serum, improved sperm vitality and testosterone levels, decreased the phosphorylation levels of NF-κB and IKKβ in the testes of mice, and played a role in inhibiting the inflammatory signaling pathway. In this experiment, we compared Metformin and 1-DNJ. The results showed that compared to the Metformin group, the DH group of mice exhibited better sperm vitality. Western blot and immunohistochemistry results also indicated that 1-DNJ had a more significant effect in reducing NF-κB phosphorylation levels than Metformin, which could be a reason for the improved sperm vitality in the DH group. However, it is worth noting that Metformin is known to have gastrointestinal side effects [60] and can lead to vitamin B12 deficiency with long-term use [61], making it difficult for some patients to adhere to long-term treatment. On the other hand, mulberry leaf tea has a long history of safe and side-effect-free use in China [20]. 1-DNJ is also considered the only internationally recognized harm-free biological product among current diabetes treatments [62]. Therefore, we believe that 1-DNJ may have advantages in improving reproductive dysfunction in obese males, especially due to its safety profile and potential to minimize side effects compared to Metformin.

## Conclusion

In summary, this study demonstrates that 1-DNJ reduces serum inflammatory factors in obese male mice, inhibits the phosphorylation levels of IKKβ and NF-κB in testicular tissue, increases testosterone levels and sperm vitality in obese male mice, and preserves the structural integrity of the testicular tissue. These findings indicate that 1-DNJ can alleviate the damage to the reproductive system of male mice caused by chronic inflammation associated with obesity. It represents a potentially effective therapeutic approach for improving the reproductive capacity of obese male mice.

## Data Availability

The data underlying this article are available in the article.
